# Dermographism

**Published:** 1893-01-28

**Authors:** 


### DERMOGRAPHISM.

An interesting account of a cutaneous affection which has only of late years received much attention, is furnished in "Le Progrès Medical" by Dr. Barthélemy. Dermographism is defined as "a condition under which the integument is able to preserve, in a very amplified and more or less durable form, the impressions which are made upon it. In a normal state firm and prolonged pressure is necessary to produce even a passing trace on the skin; here the simple contact of a soft instrument suffices to produce an impression more or less coloured, persistent, intense, and prominent." The phenomenon is more often observable among epileptic, nervous, and hysterical patients. It is sometimes due to a violent shock to the system, as in the case of a coachman who dated the extraordinary sensitiveness of his skin from a fall from his carriage. In acute cases the impression rises almost instantly upon soft pressure being applied, in the form of what is known as goose flesh, of a gradually deepening colour; the swelling increases and the line gradually extends itself, preserving the original contour for the space of about five minutes. The impression remains some hours, and then gradually dies away. The case is quoted of a young girl of sixteen, whose skin was so delicate that the kiss of a girl friend brought flaming patches to her cheeks, while the lace on her underclothing and the open work patterns of her stockings left long durable traces. This morbid condition of the skin, noted in persons of all ages, appears to be confined to no particular class or race, and is met with even among the lower animals, notably in horses.

